# Impact of mupirocin resistance on the transmission and control of healthcare-associated MRSA

**DOI:** 10.1093/jac/dkv249

**Published:** 2015-09-03

**Authors:** Sarah R. Deeny, Colin J. Worby, Olga Tosas Auguet, Ben S. Cooper, Jonathan Edgeworth, Barry Cookson, Julie V. Robotham

**Affiliations:** 1 Modelling and Economics Unit, Centre for Infectious Disease Surveillance and Control, Public Health England and Health Protection Research Unit in Modelling Methodology, London, UK; 2 Center for Communicable Disease Dynamics, Harvard School of Public Health, Boston, MA, USA; 3 Centre for Clinical Infection and Diagnostics Research, Department of Infectious Diseases, King's College London, London, UK; 4 Guy's and St Thomas' NHS Foundation Trust, London, UK; 5 Centre for Tropical Medicine and Global Health, Nuffield Department of Clinical Medicine, University of Oxford, Oxford, UK; 6 Mahidol-Oxford Tropical Medicine Research Unit, Bangkok, Thailand; 7 Division of Infection and Immunity, University College London, London, UK

## Abstract

**Objectives:**

The objectives of this study were to estimate the relative transmissibility of mupirocin-resistant (MupR) and mupirocin-susceptible (MupS) MRSA strains and evaluate the long-term impact of MupR on MRSA control policies.

**Methods:**

Parameters describing MupR and MupS strains were estimated using Markov chain Monte Carlo methods applied to data from two London teaching hospitals. These estimates parameterized a model used to evaluate the long-term impact of MupR on three mupirocin usage policies: ‘clinical cases’, ‘screen and treat’ and ‘universal’. Strategies were assessed in terms of colonized and infected patient days and scenario and sensitivity analyses were performed.

**Results:**

The transmission probability of a MupS strain was 2.16 (95% CI 1.38–2.94) times that of a MupR strain in the absence of mupirocin usage. The total prevalence of MupR in colonized and infected MRSA patients after 5 years of simulation was 9.1% (95% CI 8.7%–9.6%) with the ‘screen and treat’ mupirocin policy, increasing to 21.3% (95% CI 20.9%–21.7%) with ‘universal’ mupirocin use. The prevalence of MupR increased in 50%–75% of simulations with ‘universal’ usage and >10% of simulations with ‘screen and treat’ usage in scenarios where MupS had a higher transmission probability than MupR.

**Conclusions:**

Our results provide evidence from a clinical setting of a fitness cost associated with MupR in MRSA strains. This provides a plausible explanation for the low levels of mupirocin resistance seen following ‘screen and treat’ mupirocin usage. From our simulations, even under conservative estimates of relative transmissibility, we see long-term increases in the prevalence of MupR given ‘universal’ use.

## Introduction

Rates of healthcare-associated infection due to MRSA have fallen in many countries.^1^ Multiple interventions have been implemented to reduce the rate of MRSA infection: targeting the route of transmission, reducing the reservoir, prevention of infection arising from MRSA carriage and reducing selective pressure from antibiotic usage.^2–6^ Decolonization with antimicrobial agents such as chlorhexidine and mupirocin is a common component of MRSA-specific control strategies (with 92% of English NHS trusts using nasal mupirocin with an antimicrobial wash as the main decolonization regimen) and a landmark cluster randomized trial in ICU patients showed that ‘universal’ mupirocin usage in combination with chlorhexidine successfully reduced bloodstream infections.^7,8^ Such decolonization may reduce infections in patients through direct and indirect effects. Direct effects result from a reduction in the patient's *Staphylococcus aureus* bioburden. This is associated with a reduced risk of the patient developing a clinical infection caused by their carriage strain.^9,10^ Indirectly, reduction in *S. aureus* and MRSA bioburden may also reduce cross-transmission.^7,8^

Mupirocin has bacteriostatic activity against *Staphylococcus* through binding to isoleucyl-tRNA synthetase (*ileS*) and preventing protein synthesis.^11^ Mupirocin resistance occurs in two phenotypes: low-level mupirocin resistance (MIC between 8 and 64 mg/L) and high-level mupirocin resistance (MIC ≥512 mg/L). While high-level resistance is mediated by plasmids carrying the *mupA* gene, low-level resistance occurs through point mutations in the *ileS* gene.^12^

From evolutionary theory, an increase in mupirocin resistance in response to increasing use is expected and indeed has been reported in practice.^9,13–18^ However, the prevalence of mupirocin-resistant (MupR) MRSA strains has remained low in many settings despite widespread usage.^19–23^ The clinical and biological dynamics of *S. aureus* are complex and this has prevented the fitness cost of resistance determinants being estimated from a clinical setting to date. Furthermore, there has been no estimate to date of the relative transmissibility of MupR and mupirocin-susceptible (MupS) MRSA strains, although *in vitro* studies have suggested that, in the absence of mupirocin, there could be a fitness cost associated with MupR phenotypes.^24,25^ Previous research has estimated the transmission parameters of healthcare- and livestock-associated MRSA^26–28^ and examined the combined impact of isolation and decolonization on MRSA transmission.^26,28^ Furthermore, while mathematical modelling has been used to examine the long-term effectiveness and cost-effectiveness of competing MRSA control strategies that include the use of mupirocin,^29–32^ there has been no evaluation of the likely long-term impact of mupirocin resistance on such strategies.

We first estimate key epidemiological parameters for MupR and MupS MRSA strains using data from adult ICU and general wards (GW) collected from two large tertiary teaching hospitals. In England, both mupirocin and chlorhexidine are widely used to prevent MRSA infection.^7^ However, there are no guidelines on their usage and local infection prevention and control teams are free to recommend variable decolonization regimens as appropriate. In our setting, mupirocin was used to decolonize MRSA-positive patients in GW only. Therefore, we are able to estimate these parameters in the presence and absence of mupirocin usage.

Secondly, we use results from this analysis to parameterize an individual-based model simulating MRSA transmission, incorporating both MupR and MupS strains. We then use this model to evaluate the long-term impact of mupirocin resistance on three MRSA control policies, comparing ‘clinical cases’, ‘screen and treat’ and ‘universal’ mupirocin use. Given that mupirocin use is thought to be a key component of the current MRSA control measures, it is important to gain insight into the potential impact of resistance to such an agent.

## Methods

### Dataset

From 1 November 2011 to 29 February 2012, MRSA isolates were collected as part of mandatory screening and clinical sampling from inpatients admitted to two acute tertiary hospitals within Guy's and St Thomas' NHS Foundation Trust (GSTT). This was a subset of a larger dataset collected from inpatients and outpatients in GSTT and King's College Hospital and Lewisham NHS Foundation Trusts and community patients in Southwark, Lambeth and Lewisham London boroughs. An epidemiological description of the full dataset and details of microbiological techniques used has been reported elsewhere.^33^ MRSA isolates were submitted to the Centre for Clinical Infection and Diagnostics Research (CIDR) at GSTT. Isolates confirmed as MRSA by culture on chromogenic agar (Oxoid Brilliance) and rapid latex agglutination test (Staphaurex, Remel) were included in the study. Isolates in the dataset were screened for mupirocin resistance (low level and high level) using a modified susceptible disc breakpoint method, described in detail by Hughes *et al*.^33^ The ‘susceptible’ breakpoint was later validated by determining MICs with Etest as described.^33^

We selected wards for analysis where there were two or more patients with MRSA identified through screening. More than 95% of admitted patients had admission MRSA screening swabs collected during the study period^7^ and patients were rescreened frequently during admission, either weekly in high-risk areas or elsewhere when a ward transfer occurred or an invasive procedure was required. Seven adult ICU and 20 adult general wards (GW) in GSTT were included in our analysis. A unique anonymized patient identifier, ward name, dates of admission and discharge, were submitted with each specimen. Patient details from those screened MRSA negative were extracted from the NHS trust information system. Ward specialties and characteristics are included in Table 1 as well as numbers of MRSA-positive admission screens and acquisitions.

**Table 1.** Ward summary statistics

	Patients	Patient days	MRSA patient days	Median length of stay (days)	Observed MRSA acquisitions^a^	Observed MRSA on admission^b^
Adult ICU						
1	378	2163	11	3	0	1
2	200	2354	20	7	1	3
3	261	2487	70	5	2	2
4	161	775	45	3	1	5
5	102	340	11	2	0	3
6	49	1307	89	14	0	6
7	102	340	11	2	0	3
Adult general						
acute medicine wards	579	4609	81	5	1	6
	430	3262	19	5	3	1
cardiovascular wards	734	4207	11	3	0	6
	319	975	4	2	0	1
	754	3667	4	4	0	2
	492	4082	81	4	2	4
elderly care wards	263	4365	45	13	0	3
	256	4585	72	11	1	4
	251	4986	3	7	1	0
oncology	530	3906	65	6	1	6
	331	1057	41	3	5	5
	358	3514	39	7	1	2
surgical wards	742	3130	68	3	0	9
	783	3077	61	3	0	9
	991	2919	20	2	1	6
	332	3450	12	5	1	5
	604	3323	17	3	1	9
	764	1534	6	2	1	1
	608	4088	44	4	1	6
mixed	523	1985	11	2	1	1

^a^Patients negative on admission screen, but MRSA positive on subsequent screen.

^b^Patients tested MRSA positive from screens taken on day of admission to ward.

The MRSA decolonization protocol for patients differed between ICU and GW in GSTT. Daily octenisan or chlorhexidine skin washes were implemented for all emergency admission patients until MRSA status was known and ceased if the patient screened negative for MRSA. The need for skin washes in elective patients was based on results from MRSA screens collected in pre-admission clinics, but these patients were also screened again on admission to hospital for their elective procedure. Patients in GW who screened positive for MRSA had nasal mupirocin applied three times daily for 5 days and octenisan or chlorhexidine skin washes were continued. In the ICU, octenisan or chlorhexidine bathing was used throughout their stay and chlorhexidine also applied nasally; mupirocin was not used in ICU wards.^34^

### Parameter estimation

Using the dataset described above, we estimated the transmission rates and prevalence on admission of MupS and MupR MRSA strains.

MRSA carriage in hospital patients is always imperfectly observed because carriage is asymptomatic and can only be detected by a finite number of screening swabs, each with less-than-perfect sensitivity. To overcome this problem, we adapted a previously described data-augmented Markov chain Monte Carlo (MCMC) algorithm to estimate the key epidemiological parameters of importation and transmission from our data.^26^ The method used accounts for the resulting uncertainty surrounding patient colonization times and events.^35–38^ We grouped low- and high-level mupirocin resistance together for analysis, referring to both as MupR. We did not distinguish between asymptomatic MRSA carriers and those with signs of clinical infection. The underlying transmission model was discrete time and stochastic.^26^ As data on MSSA colonization or co-colonization were unavailable, we did not consider the impact of co-colonization of patients with MSSA or multiple MRSA strains. Additionally, we assumed that a patient colonized with MupS on admission could not become colonized with MupR through *de novo* mutation of a MupS strain during the ward stay.

Within the model, at a given timepoint each patient is assumed to be either ‘susceptible’ (MRSA negative) or ‘colonized’ (MRSA positive). Patients could be colonized with either MupS or MupR strains, but we assumed that each patient could be colonized by only one strain type at any one time.

A patient *j* enters the hospital on day $t_{j}^{\;a}$, is discharged on day $t_{j}^{\;d}$ and enters the ward with a probability *p_s_* or *p_r_* of being admitted to the ward colonized with MupS or MupR MRSA, respectively. Once colonized with MRSA of a particular strain type, the patient remains so until discharge.

The per patient transition rate for a susceptible patient to become colonized on day *t* was defined as:

$$q(t)=a_{1}C_{s}(t)+a_{2}C_{r}(t)$$

where *a*_1_ is the transmission rate for a patient colonized with a MupS strain, *a*_2_ is the transmission rate for a patient colonized with a MupR strain and *C_s_*(*t*) and *C_r_*(*t*) are the number of patients colonized with MupS and MupR strains on day *t*, respectively. The model was implemented and run in R (http://cran.r-project.org). A detailed description of this model framework and its implementation has been published previously.^26^

We estimated the transmission and importation parameters for MupS and MupR strains for each ward. A random-effects meta-analysis was used to pool the individual ward estimates, where parameter estimates for each ward were weighted by inverse variance.^39^ We split the wards into ICU and GW, as defined by the NHS trust, and estimated the mean and 95% CI for importation and transmission parameters for both MupR and MupS strains for each ward type.

When estimating transmission parameters for MupR strains, we restricted our meta-analysis to wards where there was evidence of MupR importation. We then calculated the pooled relative risk of transmission for a MupS strain, calculated as the ratio of transmission probability for a MupS versus MupR strain, *R*_MupS:MupR_. To ensure this relative risk represented the difference in transmission potential between susceptible and resistant strains, rather than differences between wards, the pooled estimate for the MupS transmission parameter (numerator) was limited to the wards from which the MupR transmission parameter (denominator) was derived.

### Individual-based model

**Figure 1.**
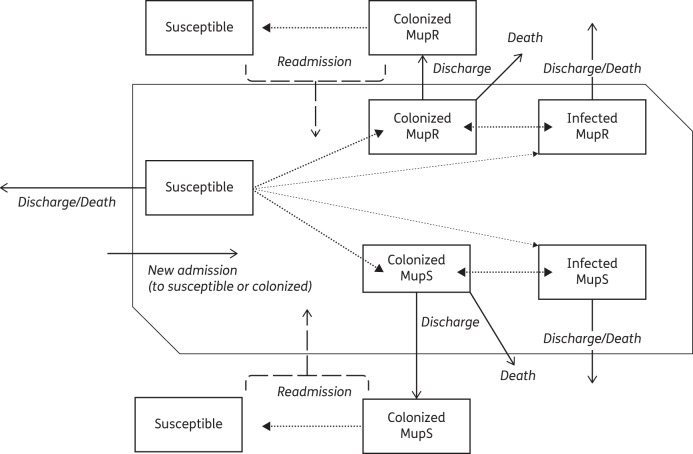
Schematic of MRSA transmission dynamics within a ward. Transitions between infection states are shown by dotted lines; a thin dotted line indicates transition unique to ICU. Continuous and dashed lines indicate patient movement as labelled in the figure.

In order to investigate the dynamics of mupirocin resistance in a healthcare facility, we developed a stochastic, dynamic, individual-based model of MRSA transmission in a whole hospital, extended to include MupS and MupR MRSA strains (Figure 1). This model was extended from one published previously: patient movement, readmission and hospital structure simulated in the model were not altered from that described in detail in the original publication.^31^ Briefly, the hospital structure was composed of two ward types (ICU and GW) and patients could transfer between these wards and be admitted, and readmitted, from the community. Length of stay, readmission and transfer parameters were determined by ward type and additionally for GW by patient specialty within the ward (general and acute care of the elderly).^31^ Patient movement, population size and hospital parameters are presented in Table 2.

**Table 2.** Patient movement parameters used in the individual-based model

	ICU	GW		Source
	all specialties	acute care of the elderly	general medical	
Daily probability of ward discharge for susceptible and MRSA-colonized patients	0.13	0.13	0.15	mean (mean and full distribution used in model described previously^31^)
Daily probability of ward discharge for MRSA-infected patients	0.08	0.09	0.12	
Daily probability of hospital discharge given a ward discharge	0.18	0.58	0.51	
Daily probability of transfer between ward types	0.56	0.0036	0.00053	
Daily probability of death for susceptible and MRSA-colonized patients	0.02	0.007	0.007	
Daily probability of death for MRSA-infected patients	0.03	0.0085		
Probability of readmission, first hospital stay	0.26	0.31	0.26	mean (mean and full distribution used in model described previously^31^)
Probability of readmission, second hospital stay	0.50	0.67	0.50	
Time (days) between discharge and readmission (mean)		96.69		
Probability that a patient will be readmitted to the same specialty	1	0.18	1	
	All specialties and wards			
Proportion of patients assigned to ‘acute care of the elderly’ specialty	0.30			
Hospital beds	ICU beds 10	GW beds 30		average ward sizes in study dataset

Each day, patients could transition between three possible states: susceptible, MRSA colonized and MRSA infected. MRSA-colonized and MRSA-infected patients could have one of two strain types (MupS or MupR) and only one strain type could colonize or infect a patient at any time. It was assumed that the probability of a susceptible patient becoming colonized with strain type MupS or MupR (in non-ICU and ICU wards) or infected directly from susceptible status (in ICU wards only, through cross-infection as previously discussed in Robotham *et al*.^29^) increased linearly with the number of MRSA-positive patients (both colonized and infected) of each strain type on their ward.

We assumed that all colonized and infected patients were equally infectious and that transmission occurred via a mass action process, i.e. all patients in the ward were equally likely to come into contact (mediated by a healthcare worker) with another patient on the ward. MRSA-colonized patients could also progress to MRSA infection through self-infection, i.e. progression from a colonized to an infected state. Although colonized and infected patients could transfer between wards, the transmission dynamics of each ward were otherwise independent.^31^ In the community, a patient could recover from MRSA colonization. However, as in previous models of hospital-associated MRSA transmission in Europe we assumed that there was no community MRSA transmission.^29,31^

Estimates derived from the MCMC analyses, as well as parameters taken from the literature, were used as the input parameters for this simulation study (Table 3).

**Table 3.** Parameters governing MRSA transmission and prevalence and mupirocin decolonization

MRSA parameters	ICU ward		GW		
	MupS	MupR	MupS	MupR	
Prevalence on admission, mean (range)	0.02 (0.01–0.1)	0.007	0.006 (0.001–0.1)	0.001	baseline value taken from meta-analysis as described (range for scenario analysis as described in the text)
Ratio of transmission: *R*_MupS:MupR_, mean (range)	2.16 (1–5)				
Daily probability of cross-colonization per source, mean (SD)	0.01 (0.017)^a^	MupS/*R*_MupS:MupR_^b^	0.0015 (0.00043)^c^	^b^	^a^MupS, estimated from data as described in the text; as indicated in the text, colonized and infected have the same contribution to transmission, thus estimated parameters from MCMC model are used ^b^value taken from Robotham *et al*.^40^; adjusted as described in the main text ^c^Robotham *et al*.^40^ ^d^assumption
Daily probability of cross-infection per source, mean (SD)	0.0006 (0.00023)^c^	^b^	0^c^	^d^	
Daily probability of progression from colonization to infection, mean (SD)	0.02 (0.0094)		0.047 (0.0094)		29
Duration of colonization, mean	365 days				41
Duration of infection	until discharge				assumption
Decolonization (mupirocin treatment for 5 days)	All wards and specialties				
	MupS		MupR		
Proportion of treated patients who are MRSA negative at treatment end, mean (SD)	0.64 (0.13)		0.27 (0.26)		9
Daily probability of reversion to MRSA-positive status for successfully treated patients	0.13				31
Proportional reduction in daily probability of progression or self-infection, mean (SD)	0.67 (0.12)^e^		0^f^		^e^van Rijen *et al*.^43^ ^f^assumption

### Interventions

We simulated three policy types: mupirocin treatment of infected MRSA cases only (‘clinical cases’); screening all patients on admission and treating identified MRSA-positive patients (‘screen and treat’); and treating all patients with mupirocin on admission, with no screening (‘universal’). The model assumed that, for the duration of mupirocin treatment, the probability of progression from a colonized to an infected state was reduced for patients colonized with MupS strains only.^42^ At the end of treatment, carriage of MupS or MupR strains was cleared with a probability drawn from a specified distribution, as described in Table 3.

### Baseline parameters

The probability of progression from MRSA colonization to infection was assumed to be equal for MupR and MupS strains and taken from the literature (Table 3).

The probability of cross-colonization (transmission probability) represents the daily probability of transmission from a single colonized or infected patient to a particular susceptible patient.

For baseline ICU specific estimates of both MupS and MupR MRSA transmission probabilities, we used the pooled ICU ward estimates from the model-based data analysis. As mupirocin was not used in ICU during the period of the study,^34^ we assumed that *R*_MupS:MupR_, the relative difference in transmissibility between MupS and MupR strains, was an estimate of the increased transmissibility of MupS in the absence of mupirocin usage.

The model-based transmission estimates from GW were complicated by the fact that mupirocin was in use in these wards during the collection of the dataset. The transmission parameters derived for MupS and MupR strains, and the relative difference between them, therefore reflected not only any underlying difference in transmissibility between these strains, but also the effectiveness of mupirocin.

To obtain an estimate of MupS transmission in GW in the absence of mupirocin usage, we used a previously derived estimate from a GW setting with MupS strains (Table 3).^40^ Under the assumption described above, that *R*_MupS:MupR_ as derived from ICU data where mupirocin was not used provided a measure of the relative difference in transmissibility between MupS and MupR strains, we used this relative difference to adjust the GW MupS estimate to provide a GW MupR transmission probability estimate (shown in Table 3).

The prevalence of MRSA in new admissions, and the proportion of those MRSA-positive patients that were MupR, *P*(MupR, import), was held constant through each 5 year simulation of the model. The MRSA status of a new patient with no previous hospitalization was assigned on admission dependent on these parameters, while the prevalence of MRSA in readmitted patients was dynamic and determined by the historical MRSA status of individual readmitted patients.

When simulating mupirocin usage in the individual-based model in either the ICU or the GW, the daily transmission probabilities of MupS and MupR strains remained the same. Mupirocin usage impacted only probability of clearance at end of treatment and reduction in probability of infection development for the duration of treatment (parameters used shown in Table 3).

### Sensitivity and scenario analyses

As there was large uncertainty surrounding our parameters estimated from the model and as data were from only two hospitals, we performed scenario analysis for *R*_MupS:MupR_ and admission prevalence of MupR as a proportion of MRSA. We therefore simulated the individual-based model with five values of *R*_MupS:MupR_ (from 1 to 5) and five values of admission prevalence *P*(MupR, import) (from 0.01 to 0.2).

To account for uncertainty in other model parameters, we performed one-way and multivariate sensitivity analyses. We assigned probability distributions derived from peer-reviewed research articles to each as described previously.^40,41^ We performed multivariate probabilistic sensitivity analysis by generating 100 parameter sets where each parameter was sampled independently and with replacement from distributions specified previously.^40,41^ We repeated this procedure 50 times, once for each combination of *P*(MupR, import) and *R*_MupS:MupR_.

To account for stochastic variation, the model was run with each parameter set 1000 times, simulating 5 years of patient dynamics, following an initial burn-in period of 1 year. After the initial burn-in period, the following initial conditions were reached: the initial prevalence of MRSA on admission was 2% and within this 2%, the prevalence of MupR on admission was 16%. MupR in the MRSA population in hospital (both in colonized and infected patients) was 3% (95% CI 2.5%–3.5%).

The model was programmed in C++ and run on a SLURM cluster.

### Simulation model output

For ‘clinical cases’, ‘screen and treat’ and ‘universal’ policies, we estimated the number of patients colonized and infected (measured in number of colonized or infected patient bed days per 10 000 bed days) with MupS and MupR MRSA over 5 years. In the first instance, we simulated these values for baseline parameters.

For both baseline and sensitivity analyses we report the statistic P(MupR)/P(MupR,import), where the numerator is the cumulative proportion of MRSA bed days due to MupR strains and the denominator is the prevalence of MupR in MRSA strains carried by patients on hospital admission. This statistic represents the excess MupR in the hospital population over and above that due to importations from the community, i.e. excess MupR arising within the hospital, which can therefore be assumed to be due to transmission. Therefore *P*(MupR)/*P*(MupR, import) is the magnitude of the increase in MupR prevalence, compared with MupR prevalence on admission. For each value of $R_{\mathrm{MupS}:\mathrm{MupR}}$ we calculated the proportion of simulations where P(MupR)/P(MupR,import)>1. Therefore describing the probability of growth of MupR prevalence within the population.^42^

### Ethics

Data used in this analysis were collected as part of research conducted following approval from the National Research Ethics Service (REC reference 11/NW/0733).

## Results

### Results from parameter estimation

**Figure 2.**
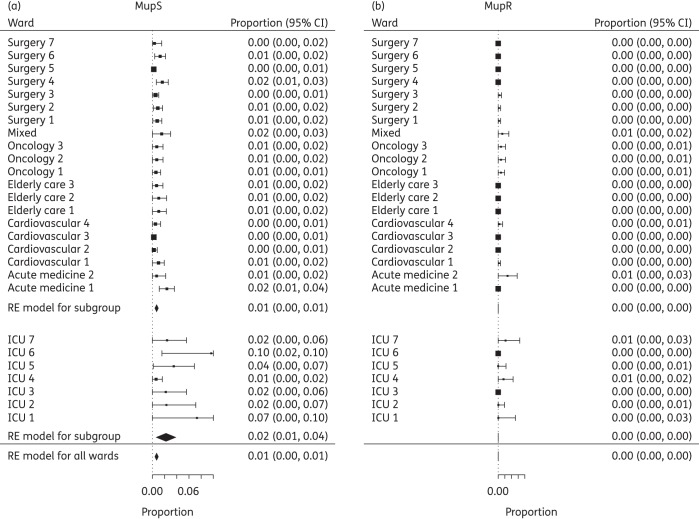
Meta-analysis of the importation probability from ICU and GW, showing the mean estimate and 95% CI as calculated by a random-effects (RE) model. Results for (a) MupS and (b) MupR strains.

The results of the meta-analyses of importation parameter estimates for adult GW and ICU are presented in Figure 2. In GW, the prevalence of MupS on admission was 0.7% (95% CI 0.5%–0.9%) of all admissions and 0.01% (95% CI 0%–0.04%) of all admissions for MupR strains. The estimated true prevalence of MRSA on admission was 2% (95% CI 0.6%–4%) of all admissions for MupS strains and 0.7% (95% CI 0.04%–1%) of all admissions for MupR strains in the ICU.

We estimated the median transmission probability per day, defined as the probability of one MRSA-negative patient acquiring MRSA given one MRSA-positive patient on the ward. In the GW, the estimated transmission probability was 0.002 (95% CI 0.002–0.004) for MupS strains and 0.005 (95% CI 0.002–0.007) for MupR strains. This was estimated at 0.01 (95% CI 0.003–0.02) for MupS strains and 0.011 (95% CI 0.0001–0.012) for MupR strains in the ICU.

**Figure 3.**
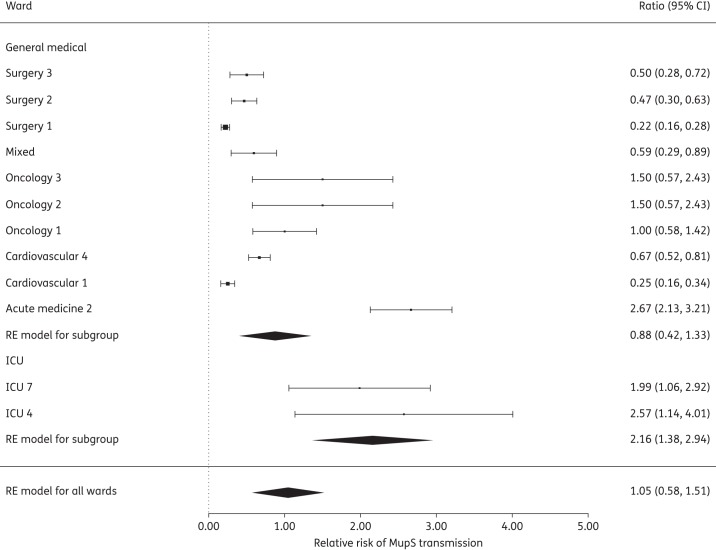
Meta-analysis of the ratio of transmission estimates for MupS/MupR strains from ICU and GW, showing the mean relative risk and 95% CI as calculated by a random-effects (RE) model.

Selecting wards where the mean proportion of MupR on admission was >0, 10 GW wards and 2 ICU wards (Figure 2b), we calculated the value of the risk ratio *R*_MupS:MupR_. *R*_MupS:MupR_ was estimated to be 2.16 (95% CI 1.38–2.94) in ICU wards and 0.88 (95% CI 0.42–1.33) in GW (Figure 3). A value >1 suggests that MupS strains have a higher probability of transmitting than resistant strains. It should be noted that mupirocin was not used in the ICU wards sampled in this study, but was used in GW on patients that tested positive for MRSA on admission.

### Simulation model results

**Figure 4.**
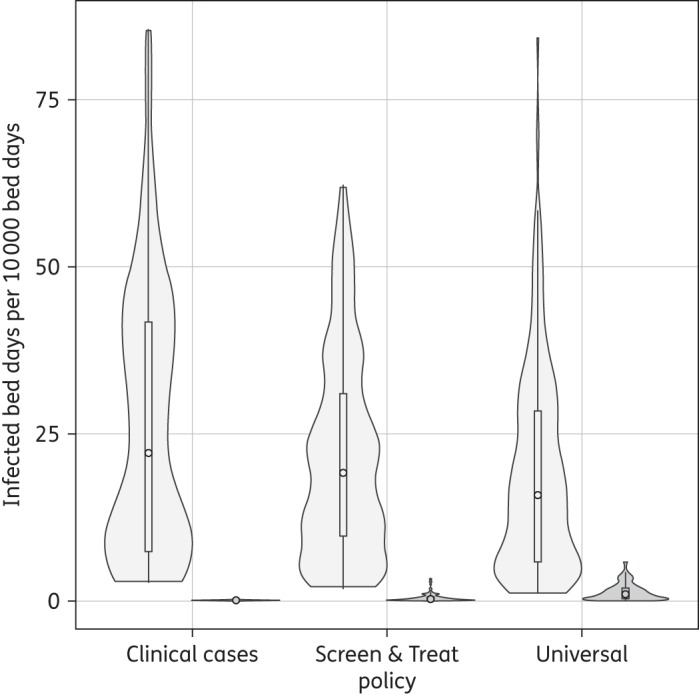
Violin plot showing the frequency distribution for number of infected bed days per 10 000 total bed days. MupS (grey) and MupR (dark grey) MRSA. The circle inside the box is the median and the bottom and top of the box are the first and third quartiles, respectively. Results are from simulations under baseline parameter assumptions as presented in Tables 2 and 3.

Treating only ‘clinical cases’ with mupirocin resulted in a median of 21 MRSA-infected bed days per 10 000 hospital bed days. ‘Screen and treat’ and ‘universal’ mupirocin usage resulted in fewer MRSA-infected bed days than the ‘clinical cases’ policy, with medians of 19 and 16 MRSA-infected bed days per 10 000 hospital bed days, respectively. Figure 4 presents the impact of each policy on MupS and MupR MRSA-infected bed days.

With ‘clinical cases’-only mupirocin usage, the total prevalence of MupR in the MRSA population in hospital (both in colonized and infected patients) was 3.8% (95% CI 3.5%–4.2%) after 5 years. With ‘screen and treat’ of those MRSA colonized on admission, the prevalence of MupR in the MRSA population in hospital (both in colonized and infected patients) was 9.1% (95% CI 8.7%–9.6%) after 5 years.

While the total number of MRSA-infected bed days decreased under ‘universal’ treatment compared with ‘screen and treat’ mupirocin usage (a percentage decrease of 4.4%; Figure 4), there was an increase in the number of MupR MRSA-infected bed days from 0.5 to 1.4 per 10 000 hospital bed days (Figure 4). The total hospital prevalence (number of MupR-infected and -colonized bed days) of MupR after 5 years increased to 21.3% (95% CI 20.9%–21.7%) under ‘universal treatment’ when compared with ‘screen and treat’.

**Figure 5.**
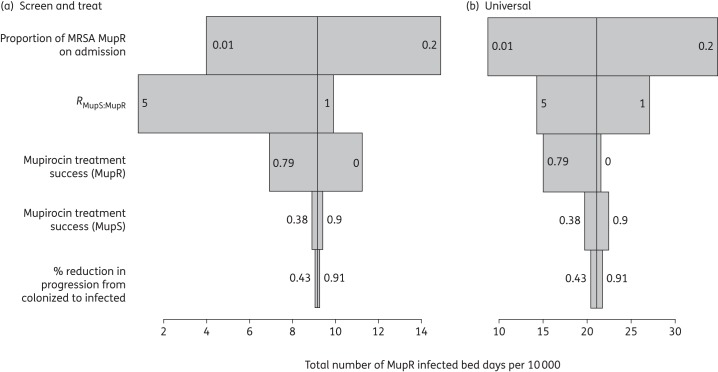
One-way sensitivity analysis showing the impact of parameter variation on the total number of MupR-infected bed days per 10 000 total bed days after 5 years of policy. Sensitivity analysis: in the plotted sensitivity analyses, only the parameters indicated in the description were varied, holding other parameter values at the base scenario (Tables 2 and 3). The maximum and minimum parameter values, corresponding to the maximum and minimum MupR-infected bed days, are indicated in text on the grey bars. Plots are shown for (a) the ‘screen and treat’ policy and (b) ‘universal’ mupirocin use.

Univariate sensitivity analysis showed that community prevalence of MupR and the relative excess transmissibility of the MupS MRSA strain had the largest impact on the number of MupR MRSA-infected bed days (Figure 5). However, the effectiveness of mupirocin in treating MupR colonization had the third largest impact on the number of MupR-infected bed days (Figure 5).

**Figure 6.**
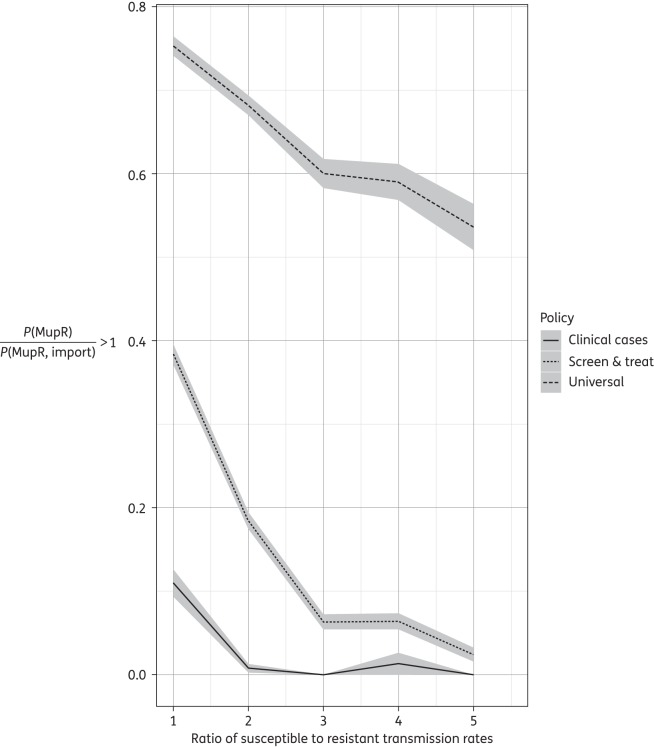
Probabilistic sensitivity analysis: probability that the prevalence of MupR exceeds the prevalence of MupR in imported MRSA after 5 years. This is measured as the proportion of simulations where, *P*(MupR)/*P*(MupR, import) > 1. *P*(MupR) is the final prevalence of MupR in MRSA colonizations and *P*(MupR, import) is the prevalence of MupR in MRSA on admission, i.e. imported. Calculated for mupirocin treatment of clinical (infected) MRSA cases only (‘clinical cases’, continuous line), MRSA-positive patients (‘screen and treat’, dotted line) and universal mupirocin usage (‘universal’, dashed line). The line is the mean of the simulations and the grey bars the 95% CI.

**Figure 7.**
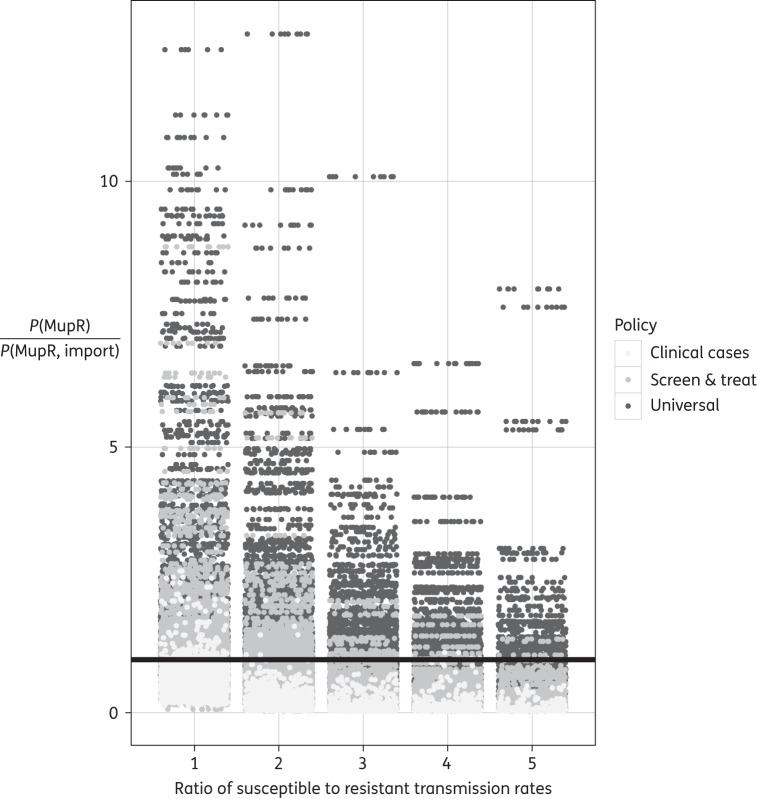
Probabilistic sensitivity analysis: ratio of prevalence of MupR in imported MRSA compared with total prevalence of MupR. Each point is the average of simulations from a parameter set. Showing the value of P(MupR)/P(MupR,import) where P(MupR)/P(MupR,import)=1 is plotted as a continuous line and indicates no change in the prevalence of MupR over time.

The probabilistic sensitivity analysis calculated the probability of an increase in MupR in the population, for the full range of parameters. Under a strategy of ‘clinical cases’ mupirocin usage, the total prevalence of MupR rarely increased. Only under conditions where MupR and MupS strains were equally infectious did MupR prevalence increase (in 10% of simulations) (Figures 6 and 7). In the case of ‘screen and treat’ mupirocin usage, when MupS strains transmitted more easily than MupR strains, MupR prevalence increased by >10% after 5 years in simulations from only nine parameter sets (Figure 6). However, when MupR and MupS strains transmitted equally well, the prevalence of MupR increased in 40% of simulations. In the case of ‘universal’ mupirocin use, when MupR and MupS strains transmitted equally well, the prevalence of MupR increased in 75% of simulations. At the other extreme, under conditions where susceptible strains transmitted five times as well as resistant strains, the prevalence of MupR strains still increased in >50% of simulations (Figure 6).

We estimated the value of *P*(MupR)/*P*(MupR, import) for each value of MupS and MupR strains (Figure 7) simulating each for the full range of other parameters. Focusing on the case where MupS strains transmit twice as well as MupR strains (the baseline parameter found in our analysis), when ‘screen and treat’ mupirocin is implemented the final prevalence of MupR was up to six times the prevalence on admission of MupR in some simulations (Figure 7). However, in the majority of cases the final prevalence of MupR did not represent an increase. In contrast, when ‘universal’ treatment was implemented the prevalence of MupR increased up to 14 times over the simulation period, though in the majority of simulations there was a <3-fold increase. This uncertainty in the outcome decreased as the relative transmissibility of the MupS strains increased.

## Discussion

From analysing samples collected from patients in two large tertiary care hospital sites, we found evidence that in the absence of mupirocin usage, MRSA strains susceptible to mupirocin transmitted more readily than those resistant to mupirocin. In the presence of mupirocin usage, this ratio was reversed. This provides evidence that MupR MRSA strains are less transmissible and therefore carry a fitness cost in comparison with MupS MRSA strains. To our knowledge, our research provides the first examination of the transmissibility of MRSA strains resistant and susceptible to mupirocin. Using these estimates, our simulation study showed that over a 5 year period with a ‘screen and treat’ policy, the prevalence of resistance in the hospital rarely increased above the prevalence on admission (Figures 6 and 7). This suggests we might reasonably expect mupirocin resistance prevalence to remain stable in the hospital population under this policy of mupirocin usage. In contrast, under ‘universal’ mupirocin usage, while MRSA infections were prevented, increases in the prevalence of MupR were likely, even under conservative estimates of relative transmissibility.

### Comparison with previous research

As this is the first study to estimate the impact of mupirocin resistance on fitness in clinical settings, we can only compare our findings with laboratory studies. While one *in vitro* study has suggested that when the strain background is similar, MRSA strains carrying *mupA* have no evidence of a difference in fitness cost,^24^ it is unlikely that all differences in transmissibility in human hosts will be reflected in laboratory fitness assays. Moreover, some *in vitro* studies have shown interactions between spontaneous mutations in the *ileS* gene and compensatory mutations. There is evidence that some spontaneous mutations conveying mupirocin resistance result in a fitness loss, which can be reversed through subsequent compensatory mutation.^25^

### Impact on long-term MRSA control

In all our baseline simulations, where MupR strains transmit less well than MupS strains, the prevalence of MupR remains low over a 5 year period. MupR strains coexist within the patient population at low levels with MupS strains. To our knowledge, this research is the first to examine the mechanisms of coexistence of MupR and MupS MRSA strains in a clinical context. However, our findings are broadly consistent with other work examining the coexistence of MRSA strains. Previous research has shown that given small differences in transmissibility and differences in antibiotic usage conditions, there can be coexistence of MRSA community-acquired and hospital-acquired strains.^44^

Mindful that the strain dynamics of MRSA are complex, we performed sensitivity analysis to show the impact of ‘universal’ mupirocin use over a wide range of MRSA transmissibility values. We show that ‘universal’ usage of mupirocin increases the probability of increasing mupirocin resistance if the transmissibility of resistant and susceptible strains is equal. Under this scenario, the prevalence of MupR increases up to 10-fold over 5 years. However, we show there is a high level of uncertainty around this estimate. This is due to uncertainty in both the transmission values and in the other parameters (as shown in the univariate sensitivity analysis) and results in a difference of up to 10 bed days per 10 000 patient bed days. This is greater than the difference in the reduction in infected MRSA bed days, which was 6 days through ‘universal’ mupirocin usage. We believe this provides further evidence that policies encouraging ‘universal’ mupirocin usage should be approached with caution and accompanied by surveillance for mupirocin resistance. Moreover, consideration of the cost of mupirocin resistance should be included in any health economic evaluation of intervention strategies involving decolonization.

### Limitations

It was not possible to examine low- and high-level mupirocin resistance separately, because of the low prevalence of MRSA in the hospitals sampled. Likewise, further research is needed to fully examine any role of co-colonization with other staphylococcal species in the development and transfer of mupirocin resistance. Our results show an association between reduced transmission and MupR MRSA strains. Examining each MRSA ST separately would represent an important next step. Molecular analysis of MRSA isolates in this dataset^33^ showed that mupirocin resistance was predominant in only a limited number of STs (ST36, ST8 and ST239/241), but rare in the dominant UK MRSA clone (ST22) or sporadic MLSTs. This suggests that changes in MRSA clonal epidemiology may also play a role in determining the long-term prevalence of mupirocin resistance. However, the small sample size in our dataset would not have allowed us to achieve appropriate statistical power when estimating transmission and importation parameters for MupR and MupS MRSA for each ST. Further research is needed to examine the impact of clonal differences on the relative transmissibility of MupR strains.

Due to small sample size, we have also not considered the impact of reduced susceptibility to chlorhexidine or other antibiotics in some strains. There is some evidence that when combined with carriage of antiseptic resistance genes (*qacA/B*), MupR strains are harder to eradicate with decolonization protocols.^13^ However, the relationship between carriage of *qacA/B*, reduced chlorhexidine susceptibility and reduced eradication after treatment is still uncertain and was not considered in our model.^45^ Likewise, the role of ‘bystander’ selection of MupR MRSA strains through the usage of other antibiotics may also play a role in determining the long-term prevalence of mupirocin resistance, but was beyond the scope of our analysis.

There remains uncertainty surrounding the biological action of mupirocin on MRSA transmission and the extent to which reducing the bioburden of MupS MRSA colonization reduces onward transmission from colonized patients. When simulating the long-term impact of mupirocin resistance on MRSA transmission, we conservatively assumed that there was no reduction in transmission from MupS-colonized patients when treated with mupirocin. This transmission model structure is consistent with previous models including mupirocin effect.^10,42^

As shown in the univariate sensitivity analysis, the prevalence of MupR in the community was the primary driver of the final number of MupR infections in the modelled hospital. We assumed, as in previous models of MRSA transmission in Europe, that there was no onward MRSA transmission in the community.^29,31,32,46^ We did not consider the influence of the *de novo* development of resistance in patients after treatment or the influence of patient transfers from settings of high mupirocin resistance prevalence, which may play a role in determining the higher mupirocin prevalence evident in our dataset compared with the larger south London sample.^33^ There has been evidence of mupirocin resistance in community-acquired strains^47–49^ and in settings with high community mupirocin usage and MRSA transmission; these may be important factors in driving the spread of MupR strains. We also were not able to consider the impact of readmission and ward transfer in the parameter estimation model. With the short length of ward stay, this may have resulted in underestimation of transmission events and underdetection of mupirocin resistance. However, such analysis would be beyond the scope of the dataset. Such advances on the model structure are beyond the evidence base at this time, but should be areas of future research or prioritized in countries where such an issue is already apparent.

### Conclusions

In this paper, we add to evidence that MupS strains are more transmissible than MupR strains. This may help explain the limited increase in mupirocin resistance seen despite increasing usage in some settings;^8^ however, we urge caution with implementing policies of widespread mupirocin usage. From our simulations, even under conservative estimates of relative transmissibility, we see long-term increases in the prevalence of MupR with universal use. Our models could be extended to assess transmissibility of different MRSA clones and simulate their long-term dynamics under different control strategies.

## Funding

The research outlined here is part of the Infection Theme Programme of the National Institute for Health Research (NIHR), Collaboration for Leadership in Applied Health Research and Care South London, at King's College Hospital NHS Foundation Trust.

S. R. D. was supported by the UK National Institute for Health Research Health Protection Research Unit (NIHR HPRU) in Modelling Methodology at Imperial College London in partnership with Public Health England (PHE) (grant number HPRU-2012-10080). C. J. W. received support from the National Institute of General Medical Sciences of the National Institutes of Health under award number U54GM088558. O. T. A. and J. E.: Guy's and St Thomas' NHS Foundation Trust/King's College London comprehensive biomedical research centre. B. S. C. is supported by the Medical Research Council and the Department for International Development (grant number MR/K006924/1). The Mahidol-Oxford Tropical Medicine Research Unit is supported by the Wellcome Trust of Great Britain (grant number 106491/Z/14/Z and 089275/Z/09/Z). B. C. and J. V. R. carried out this study as part of their routine work and have no relevant additional funding to declare.

## Transparency declarations

None of the authors has any financial conflicts of interest to declare. The funding bodies had no role in study design, data collection and analysis, decision to publish or preparation of the manuscript.

## Disclaimer

The views expressed are those of the authors and not necessarily those of the NHS, the NIHR, the Department of Health or PHE.
